# Synthesis and Evaluation of 8-Aminoquinoline-Grafted Poly(glycidyl methacrylate) for the Recovery of Pd(II) from Highly Acidic Aqueous Solutions

**DOI:** 10.3390/polym10040437

**Published:** 2018-04-14

**Authors:** Bing Zhang, Shixing Wang, Likang Fu, Libo Zhang

**Affiliations:** 1 State Key Laboratory of Complex Nonferrous Metal Resources Clean Utilization, Kunming University of Science and Technology, Kunming 650093, China; 15198750002@163.com (B.Z.); fulk324@163.com (L.F.); 2 Faculty of Metallurgical and Energy Engineering, Kunming University of Science and Technology, Kunming 650093, China; 3 Key Laboratory of Unconventional Metallurgy, Ministry of Education, Kunming 650093, China

**Keywords:** adsorption, palladium, efficiency, selectivity, reusability

## Abstract

A new adsorbent was prepared via modified poly(glycidyl methacrylate) with 8-aminoquinoline (AQ-PGMA) for the recovery of Pd(II) from solution. The practical application values of AQ-PGMA, including efficiency, selectivity and reusability for the recovery of Pd(II), are proved by the various experiment parameters. The parameters include HCl concentration, adsorption time, initial Pd(II) concentration, coexisting ions and reused cycles. The prepared AQ-PGMA showed a high adsorbing capacity for Pd(II) (up to 267.90 mg/g) when the concentration of HCl is higher than 0.4 mol/L. The analysis of the adsorption process indicated that the adsorption kinetics followed a pseudo-second-order kinetic model and the adsorption isotherms obeyed the Hill model. The Hill model showed that one adsorption site on the AQ-PGMA could combine 1.45 Pd(II). In addition, the obtained adsorbent demonstrated good regenerative ability and satisfying selectivity for the recovery of Pd(II). The adsorption mechanism was dominated by the chelation and ion exchange reactions between amines/hydroxyl groups and Pd(II). The experiments confirmed that AQ-PGMA was efficient for recovery of Pd(II) from highly acidic aqueous solutions.

## 1. Introduction

Palladium has been widely utilized in various fields, including electronics, fuel cells, catalysts and medicine, due to its attractive phy-chemical characteristics [1,2]. In recent years, the demand for palladium has been increasing while the high-quality natural reserves continue to be exhausted [3]. In addition, it will inevitably lead to an increase of palladium waste-water with the expansive application of palladium. So, it is necessary to develop a new technique for the recovery of palladium from the waste-water for the perspective of resource recovery and sustainable chemical industries.

Many techniques, including co-precipitation [4], ion exchange [5,6], solvent extraction [7], liquid membranes separation [8] and adsorption [9,10], have been used to recover palladium(II) from leachates, plating wastewaters and other aqueous solutions. Among these techniques, adsorption has attracted more and more attention in recent years because it is low cost and relatively simple. Generally, the adsorbents include metal-organic frameworks [11], active carbon [12,13], minerals [14], ion exchange resin [15], microcapsules containing a metal ligand [16,17,18,19], and so on. However, many adsorbents have the low capacity and poor selectivity in acidic media because leachates, plating wastewaters and other aqueous solutions containing palladium are very acidic. Therefore, it is very important to explore new adsorbents to apply directly to wastewaters without chemical neutralization.

Poly(glycidyl methacrylate) (PGMA) has received more attention due to its distinctive acid and base resistance and mechanical strength [20,21]. In addition, PGMA can be directly functionalized with other groups for a variety of applications because it has the abundant reactive epoxy group [22,23]. Hence, PGMA based adsorbents show a good prospect in the industrial application.

In this work, a novel adsorbent (AQ-PGMA) was prepared via functionalizing poly(glycidyl methacrylate) (PGMA) with 8-aminoquinoline for the recovery of Pd(II) from solution. In order to evaluate the practical application value of AQ-PGMA, the experimental parameters, including HCl concentration, adsorption time, initial Pd(II) concentration, coexisting ions and reused cycles, were investigated. Moreover, kinetics, isothermal process and mechanism of adsorption have also been studied.

## 2. Experimental

### 2.1. Materials

Glycidyl methacrylate (GMA), azobisisobutyronitrile (ABIN), polyvinyl pyrrolidone (PVP), anhydrous ethanol (99%), 8-aminoquinoline and acetic acid were purchased from Aladdin Chemistry Co. Ltd., Shanghai China. ZnCl_2_ (99.5%), CoSO_4_·7H_2_O (99.5%), NiSO_4_·6H_2_O (98.5%) and PbCl_2_ (99.5%) were obtained from Tianjin Chemical Reagent Co. Ltd., Tianjin, China. Palladium(II) solution is prepared using PdCl_2_ dissolved in 12 mol/L HCl. The acidity of the solution is controlled by HCl.

### 2.2. Synthesis of 8-Aminoquinoline-Grafted Poly(Glycidyl Methacrylate) (AQ-PGMA)

**Scheme 1:**
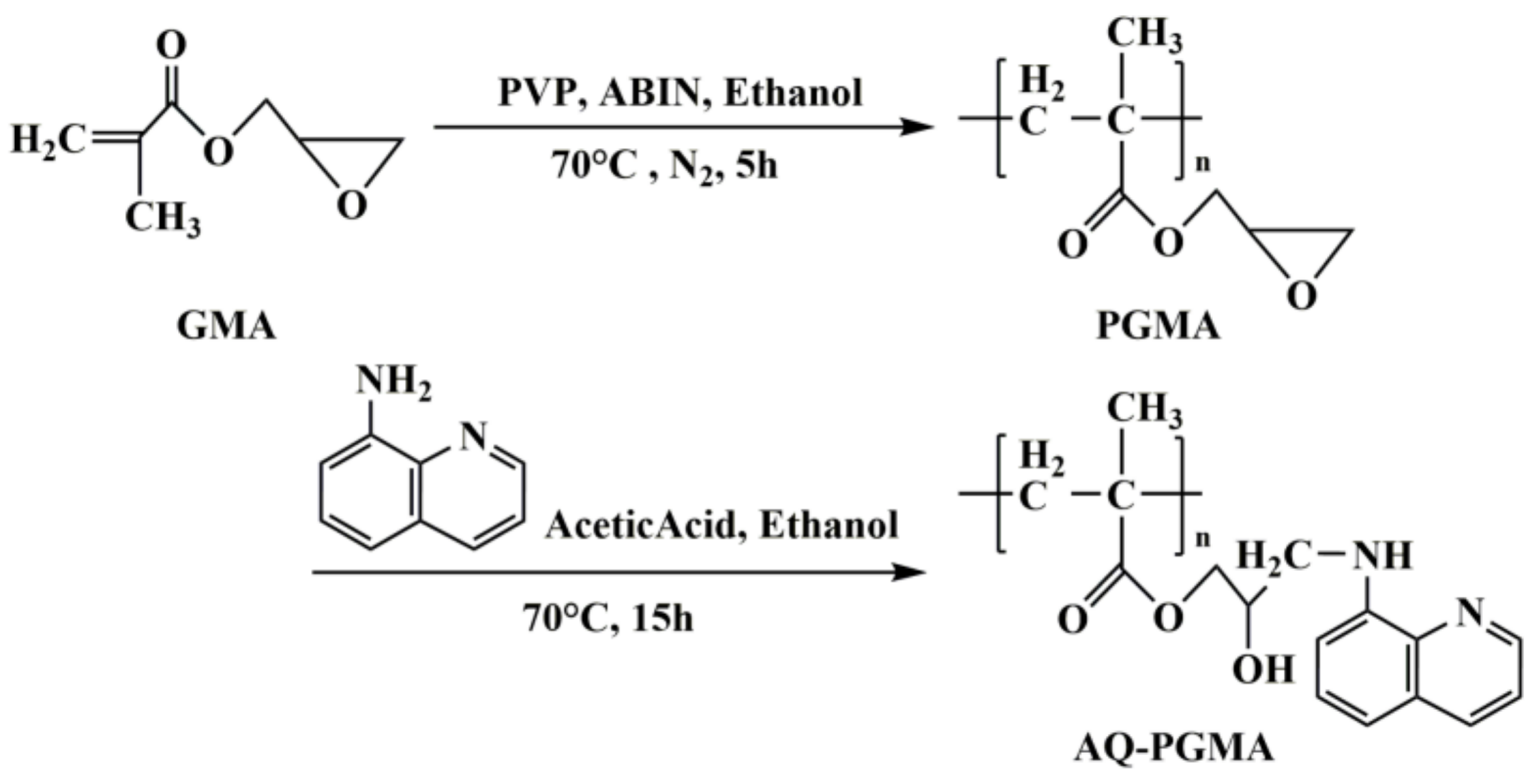
The synthesis route of 8-aminoquinoline-grafted poly(glycidyl methacrylate).

The synthesis route of 8-aminoquinoline-grafted poly(glycidyl methacrylate) (AQ-PGMA) is shown in Scheme 1. Firstly, GMA (10.0 g), PVP (3.0 g) and ABIN (0.12 g) were added to 150 mL ethanol in flask and reacted for 5 h at 70 °C with continuous stirring under N_2_. After being washed with ethanol several times, the solid product (PGMA) was dried under vacuum at 50 °C for 24 h. Secondly, a mixture of PGMA (5.0 g), 8-aminoquinoline (5.0 g), acetic acid (2 mL) and ethanol (100 mL) was reacted for 15 h at 70 °C with continuous stirring. After being washed, filtered and dried, the AQ-PGMA was obtained.

### 2.3. Adsorption Experiments

Experimental parameters including HCl concentration, adsorption time, initial Pd(II) concentration and reused cycles, were used to investigate the adsorption process and evaluate the practical application value of AQ-PGMA. In a typical adsorption experiment, 10 mg of AQ-PGMA was added to Pd(II) solution with a certain concentration and shaken for a certain time at 298 K and 250 rpm in an orbital shaker. After being separated by centrifuge, the supernatant and solid products were analyzed. The mathematical expression of the adsorption rate R (%) and equilibrium adsorption capacity *q*_e_ (mg/g) are defined as Equations (1) and (2), respectively.

(1) $$R\%=\frac{\left(C_{0}-C_{e}\right)}{C_{0}}\times 100\%\;$$

(2) $$q_{e}=\frac{\left(C_{0}-C_{e}\right)}{m}V\;$$

where *C*_0_ (mg/L) stands for the initial concentration of Pd(II), *C*_e_ (mg/L) is the concentration of Pd(II) after the process of adsorption. *V* (L) is the volume, and *m* (g) is the mass of AQ-PGMA.

### 2.4. Characterization

Fourier transform-infrared (FT-IR) spectroscopy was recorded with a Nicolet iS50 (Thermo Scientific Co., Waltham, MA, USA). The X-ray photoelectron spectroscopy (XPS) was detected with a VG Scientific ESCALab220I-XL (Thermo Scientific Co., Waltham, MA, USA). The microstructure of the samples was characterized by scanning electron microscope (SEM, Phenom ProX, Royal Dutch Philips Electronics Ltd., Amsterdam, The Netherlands). The concentrations of metal ions were detected with an inductively coupled plasma optical emission spectrometer (ICP-OES, Leeman Labs Prodigy7, Hudson, NH, USA). The surface characteristics are analyzed with a surface and porosity analyzer (Micromeritics, ASAP 2020, Micromeritics Instrument Corp., Norcross, GA, USA).

## 3. Results and Discussion

### 3.1. Characterization of AQ-PGMA

**Figure 1:**
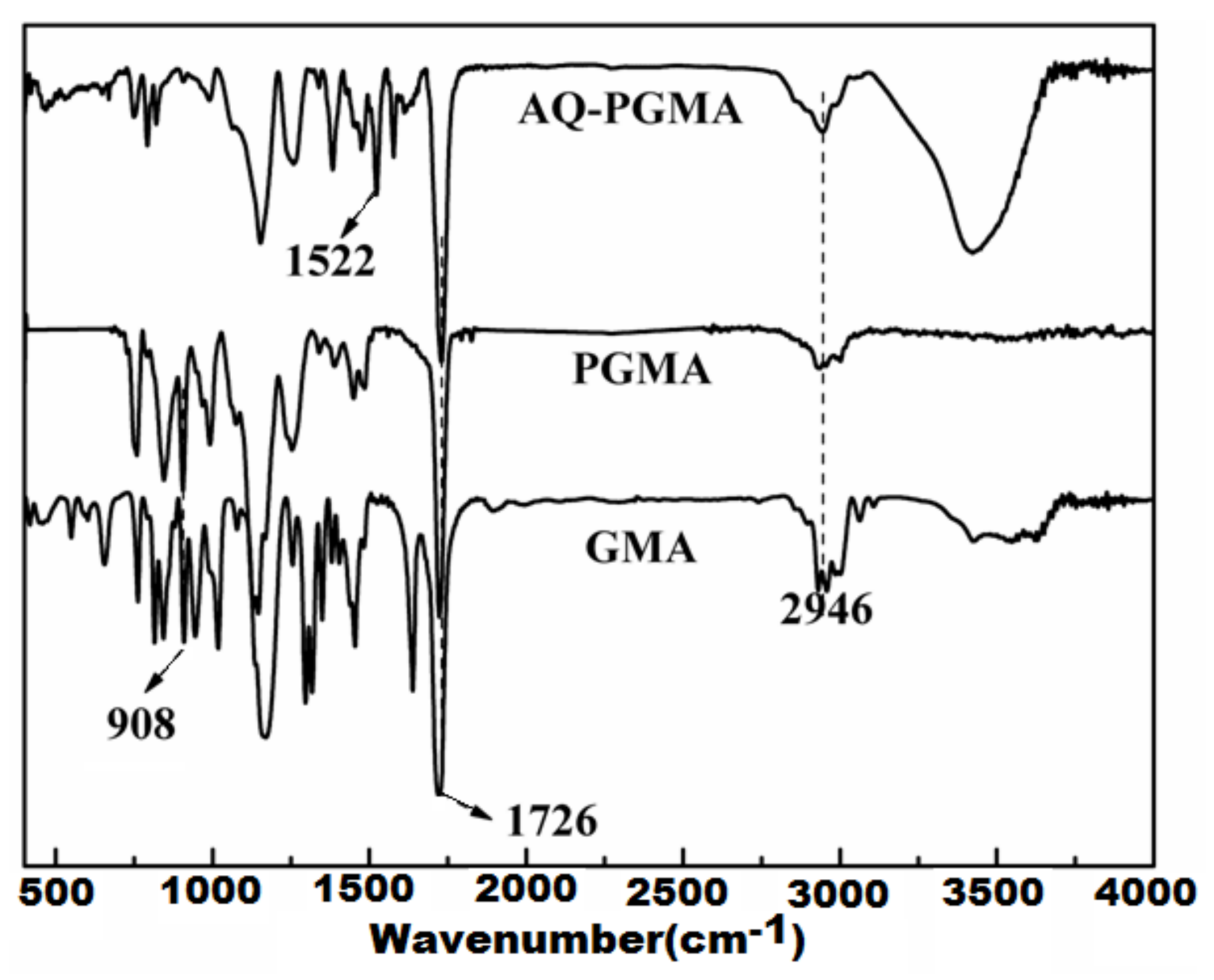
Fourier transform-infrared (FT-IR) spectra of glycidyl methacrylate (GMA), poly(glycidyl methacrylate) (PGMA) and 8-aminoquinoline-grafted poly(glycidyl methacrylate) (AQ-PGMA).

Figure 1 presents the FT-IR spectra of GMA, PGMA and AQ-PGMA. For PGMA, some peaks appear at 2946, 1726 and 908 cm^−1^, which are related to the –CH_2_ vibration peak, the C=O vibration peak and the epoxy group vibration peak, respectively [24,25]. After grafting 8-aminoquinoline, the peak of the epoxy group at 908 cm^−1^ disappeared and a new peak of pyridine at 1522 cm^−1^ appeared [17].

**Figure 2:**
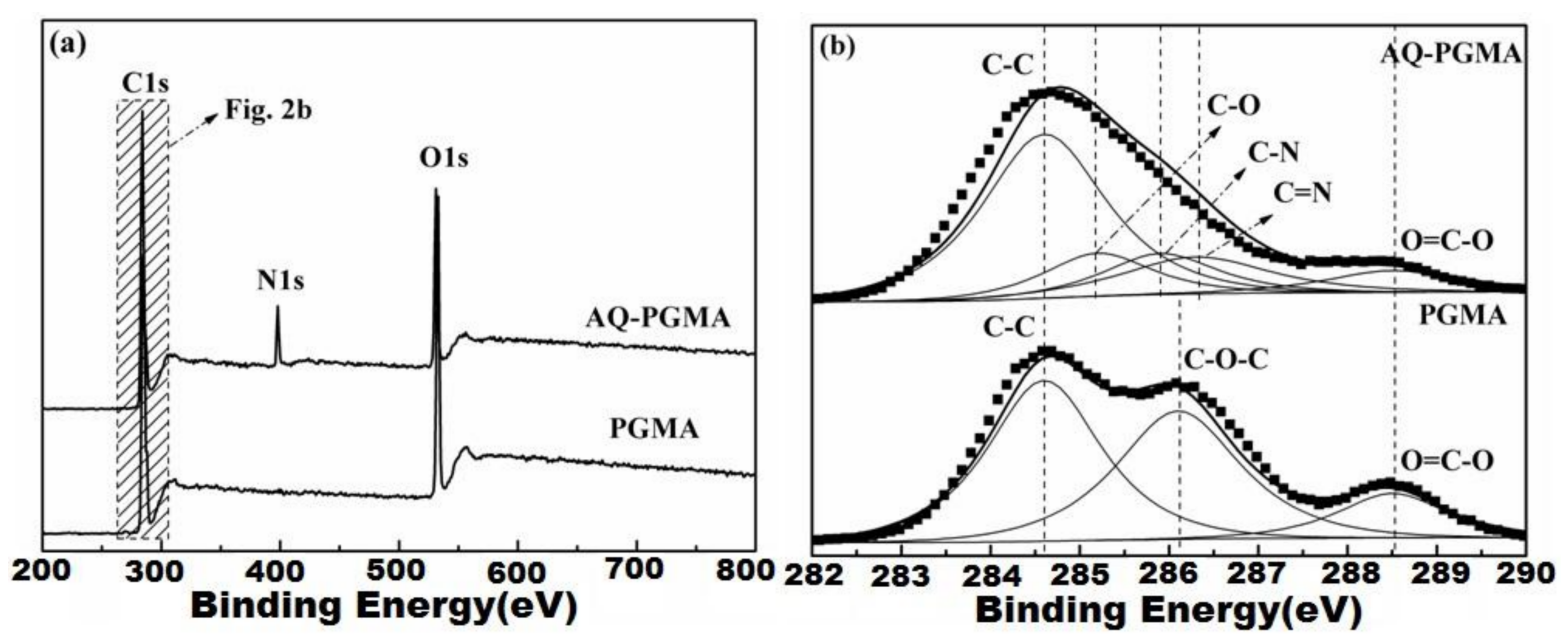
X-ray photoelectron spectroscopy (XPS) survey scan (**a**) and C 1s spectra (**b**) of PGMA and AQ-PGMA.

Figure 2a presents the XPS survey scan of PGMA and AQ-PGMA. A new peak of N 1s appears in AQ-PGMA compared to the PGMA. Figure 2b shows the C 1s spectra of PGMA and AQ-PGMA. In PGMA, the functional groups of C–O–C and O=C–O are located at 286.1 and 288.5 eV, respectively [26,27,28]. After being modified by 8-aminoquinoline, the peak of C–O–C in PGMA disappears. Meanwhile the new peaks of C–O (285.2 eV), C–N (285.9 eV) and C=N (286.3 eV) are found in AQ-PGMA [26,27,28]. Thus, 8-aminoquinoline was introduced onto PGMA and the AQ-PGMA was successfully prepared.

### 3.2. Effect of Experimental Parameters on Pd(II) Adsorption by AQ-PGMA

In order to investigate the adsorption process and evaluate the practical application value of AQ-PGMA in the recovery of palladium(II) from solution, the effects of HCl concentration, adsorption time, initial Pd(II) concentration, coexisting ions and reused cycles were investigated.

#### 3.2.1. Effect of the HCL Concentration on Pd(II) Adsorption

**Figure 3:**
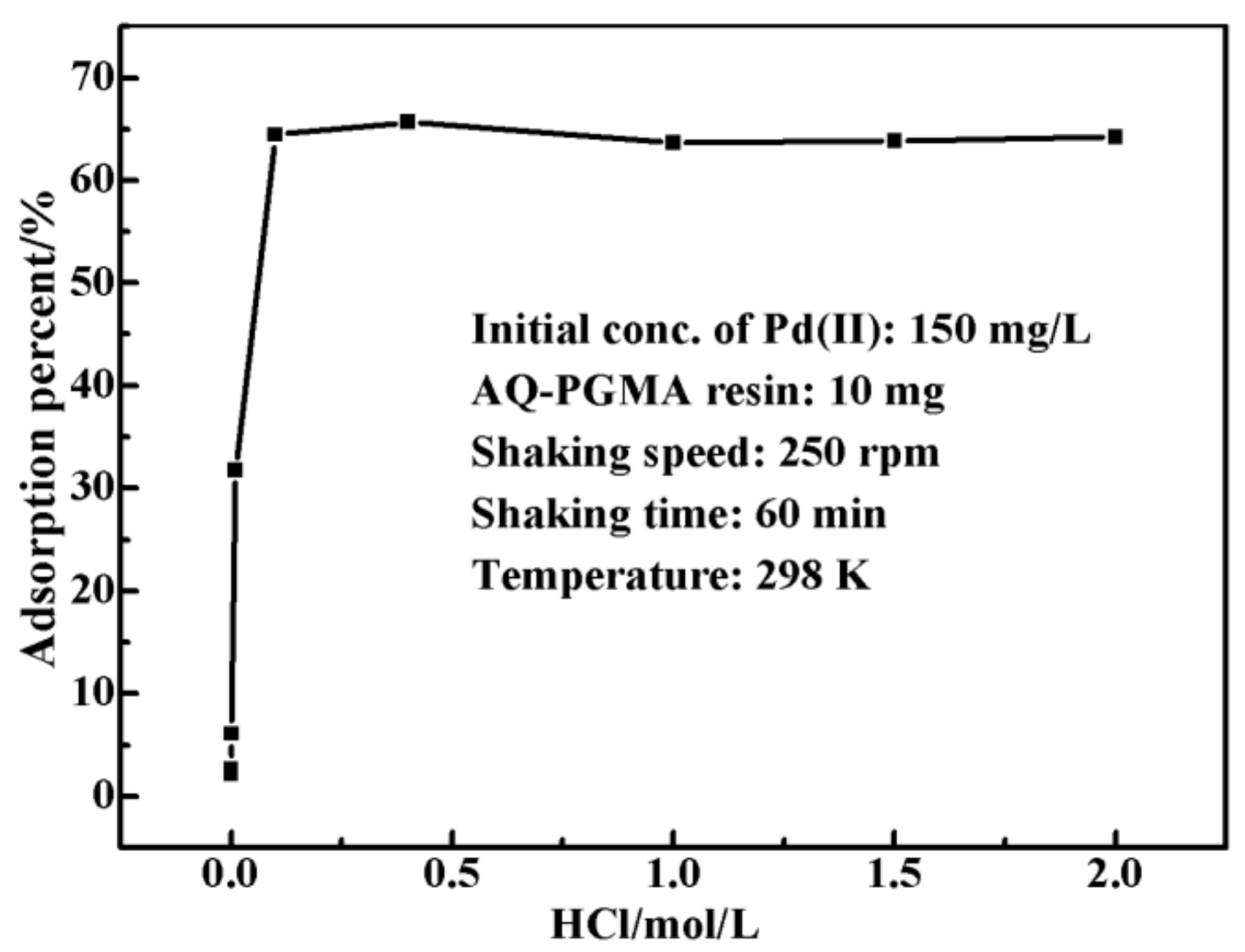
Effect of the HCl concentration on the adsorption of Pd(II) by AQ-PGMA.

It has been demonstrated that the electrostatic adsorption or complexation reactions are observably affected by the acid concentration [29,30,31,32]. In order to investigate the effect of the acid concentration on the adsorption of Pd(II) by AQ-PGMA, the mixtures of 10 mg of AQ-PGMA and 15 mL Pd(II) (150 mg/L) solution in different concentrations of HCl were stirred for 60 min at 250 rpm, and the results are shown in Figure 3. The adsorption percent of Pd(II) obviously increases from 2.14% to 65.67% with an HCl concentration range from 0 to 0.4 mol/L. In addition, when the HCl concentration is higher than 0.4 mol/L, the adsorption percentage is still higher than 65.67%. Previous studies have confirmed that the adsorption percent of palladium onto the resin particles depend on the distribution of the Pd(II) species [33]. The dominating species of Pd are PdCl_3_^−^ or PdCl_4_^2−^ when the concentration of Cl^−^ is 0.01 mol/L or over. Moreover, the enhancement of the HCl concentration promotes the protonation of AQ-PGMA and the positive charge on the surface. So, the ion exchange is one of the mechanisms of adsorption. The leaching solution of palladium is strongly acidic. The AQ-PGMA can be applied to the recovery of palladium ions from highly acidic solutions with a low spend of chemical agent.

#### 3.2.2. Effect of Adsorption Time on Pd(II) Adsorption and Adsorption Kinetics

**Figure 4:**
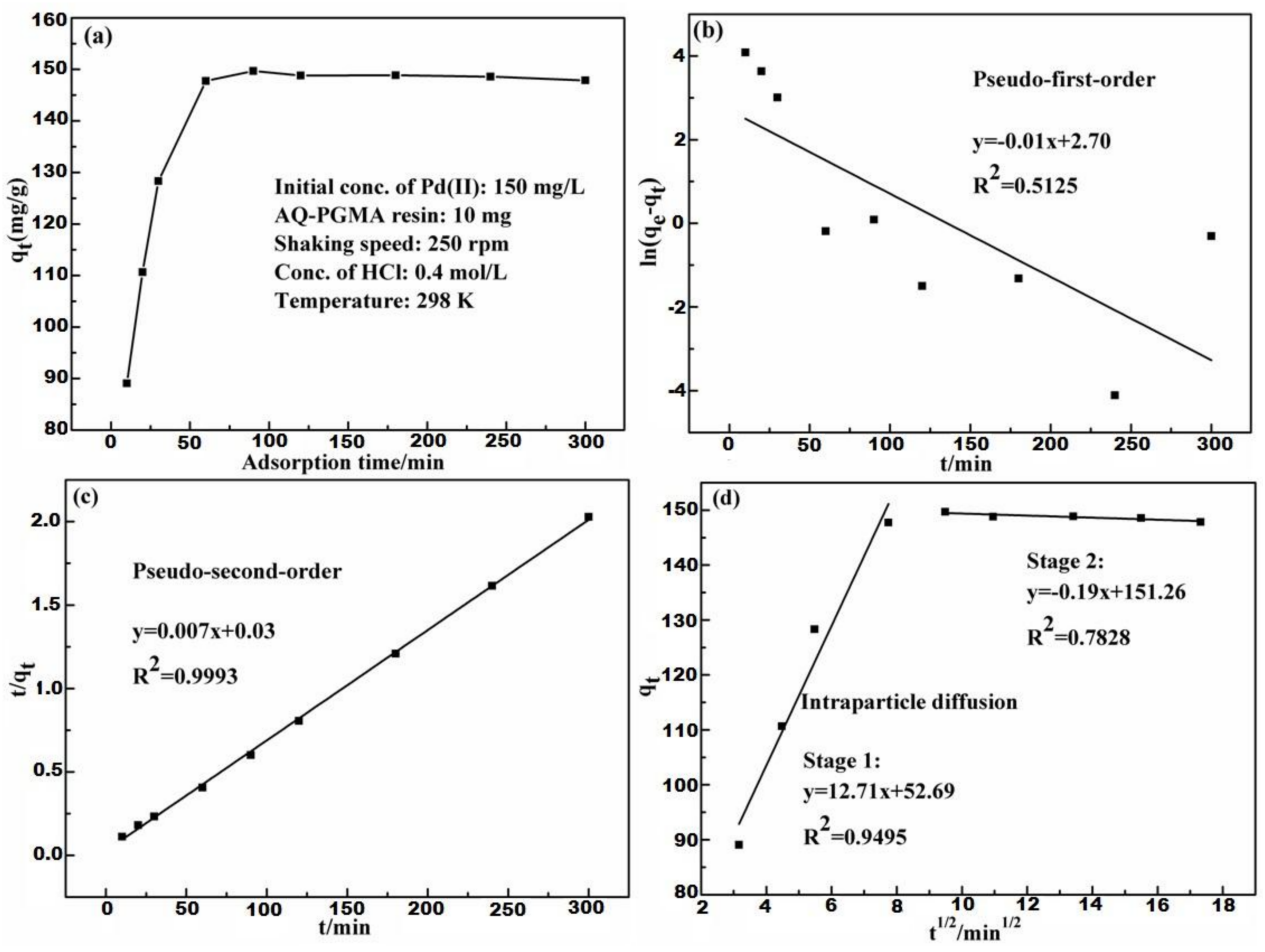
Effect of adsorption time on the adsorption of Pd(II) by AQ-PGMA (**a**) and kinetic models (Pseudo-first-order (**b**), Pseudo-second-order (**c**), Intra-particle diffusion (**d**)).

Figure 4a shows the effect of adsorption time on Pd(II) adsorption when the initial concentration of Pd(II) is 150 mg/L, AQ-PGMA is 10 mg and HCl concentration is 0.4 mol/L. The absorption rate of Pd(II) by AQ-PGMA is augmented with the lapses of the adsorption time. The increase of *q*_t_ for Pd(II) is quick in the initial phase and slows down with the lapses of the adsorption time. The result shows that the adsorption process can reach equilibrium within 60 min.

The kinetic models of Pseudo-first-order, Pseudo-second-order and Intra-particle diffusion were used to analyse the kinetics process of adsorption of Pd(II) by AQ-PGMA. The required data for fitting are taken from Figure 4a and the equations of kinetic models are expressed in Table 1 [34,35,36].

Where, *q*_e,1_ and *q*_e,2_ (mg/g) stand for the adsorbed capacity of Pd(II) by AQ-PGMA for Pseudo-first-order and Pseudo-second-order models at equilibrium. *q*_t,1_ and *q*_t,2_ (mg/g) are the adsorbed amounts at time *t* (min) for the Pseudo-first-order and Pseudo-second-order model. *k*_1_, *k*_2_ (g/(mg·min)) and *k*_i_ (mg/(g·min^1/2^)) are the rate constants of the Pseudo-first-order, Pseudo-second-order and Intra-particle diffusion models, respectively. *C* is a constant relative to the thickness of the liquid film.

The fitting results and parameters of the kinetic models on the adsorption of Pd(II) by AQ-PGMA are shown in Figure 4b–d and Table 2. Obviously, compared to the Pseudo-first-order model, the Pseudo-second-order model is the most suitable model for explaining the adsorption process, since the *R*^2^ value is closest to the unity and the calculated *q*_e,2_ value (151.29 mg/g) is approximate to the experimental data *q*_e_ value (148.60 mg/g). The results of the experiment demonstrate that the adsorptive behavior of Pd(II) on AQ-PGMA is analogous to the chemical adsorption, incorporating the interaction between the captured Pd(II) and the exposed functional groups of AQ-PGMA. Two linear regions in the fitting result of the intra-particle diffusion model divide the adsorption process into 2 stages. The slope in the first stage is the largest, and is ascribed to the move of Pd(II) from the solution to the surface of AQ-PGMA under film diffusion. The slope of the second stage is smaller, corresponding to the equilibrium stage. Both lines of regions 1 and 2 do not go through the point (0,0) and *C*_1,_ *C*_2_ ≠ 0 indicates the adsorption process is co-limited by the liquid film and intraparticle diffusion.

Table 3 shows the equilibrium time and kinetic models rate constant (*k*) of AQ-PGMA and other adsorbents in the literature. It can be found that AQ-PGMA has a high absorption rate and rate constant, which indicate that the AQ-PGMA possesses good adsorption properties.

#### 3.2.3. Effect of Initial Pd(II) Concentration on Pd(II) Adsorption and Adsorption Isotherms

**Figure 5:**
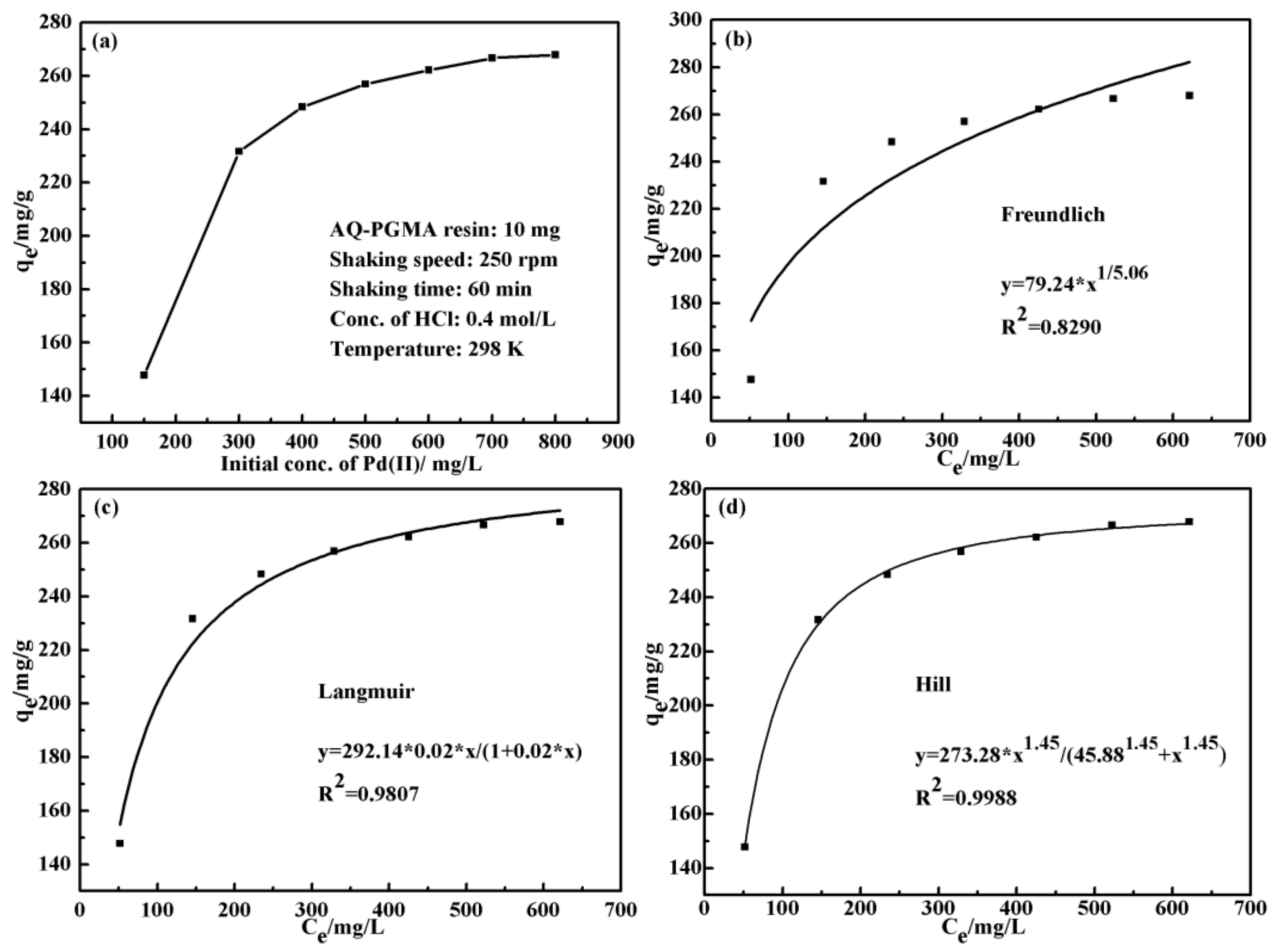
Effect of initial Pd(II) concentration on the adsorption of Pd(II) by AQ-PGMA (**a**) and adsorption isotherm models (Freundlich (**b**), Langmuir (**c**), Hill (**d**)).

To study the effect of the initial concentration of Pd(II) on the adsorption of Pd(II) by AQ-PGMA, 10 mg of AQ-PGMA was added to the different initial concentration of Pd(II) solution when the HCl concentration was 0.4 mol/L. The mixture was shaken for 60 min at 150 rpm and 298 K and the result is shown in Figure 5a. It is well known that the higher initial concentration of Pd(II) is a favorable condition for mass transfer [37]. The adsorption capacity (*q*_e_) of Pd(II) increased sharply to 260.11 mg/g when the initial concentration of Pd(II) reached to 600 mg/L. The *q*_e_ increases slowly with the further enhancement of initial Pd(II) concentration. The prepared AQ-PGMA possesses a high adsorbing capacity for palladium(II) (up to (267.90 mg/g). This result shows that the AQ-PGMA can be used for the sorption of Pd(II) from solutions.

The adsorption isotherm models, such as Freundlich, Langmuir and Hill, are employed to explore the reason for the adsorption of Pd(II) by AQ-PGMA. The Freundlich model is an empirical model and depicts the heterogeneous adsorption system and the equation is given by: [38]

(3) $$q_{e}=K_{F}C_{e}^{1/n_{F}}$$

where, *q*_e_ (mg/g) and *C*_e_ (mg/L) represent adsorption capacity and concentration of Pd(II) at equilibrium time, respectively. *K*_F_ and *n*_F_ are constants without any significance of physical chemistry.

The Langmuir model assumes that the adsorption is monolayer adsorption, all the adsorption sites are similar and that there is no interaction effect between adsorbates. The equation of the Langmuir model is expressed by: [39,40]

(4) $$q_{e}=\frac{q_{m}K_{L}C_{e}}{1+K_{L}C_{e}}$$

where, *q*_m_ (mg/g) is the maximum adsorption capacity of Pd(II), *K*_L_ is a constant relevant to binding energy in the process of adsorption. 

The Hill model is based on the grand canonical ensemble and assumes that the adsorbates form a layer on the surface of the adsorbent. The Hill model can be expressed by: [41,42]

(5) $$q_{e}=\frac{nN_{M}C_{e}^{n}}{C_{e}^{n}+C_{1/2}^{n}}=\frac{q_{0}}{1+{\left(\frac{C_{1/2}}{C_{e}}\right)}^{n}}$$

where, *N*_M_ (mg/g) stands for the density of the receptor sites on the adsorbent and *n* is the number of Pd(II) on per site, *q*_0_ is the saturated adsorption quantity of Pd(II) and *C*_1/2_ represents the adsorption concentration of Pd(II) at half saturation.

The fitting results and relevant parameters are shown in Figure 5b–d and Table 4. Figure 5b–d shows that the adsorption isotherms follow the Hill model, because the correlation coefficient (*R*^2^) is closest to 1. The saturated adsorption quantity can be reached to 273.28 mg/g according to the calculated *q*_0_ in Hill model, which is close to the experiment data (267.90 mg/g). The *n* is 1.45, suggesting that the number of Pd(II) on per site is 1.45, which also can explain why the adsorption of Pd(II) by AQ-PGMA is efficient. The comparison of AQ-PGMA with other adsorbents is shown in Table 5, it can be seen that the maximum adsorption capacity of AQ-PGMA exceeded many adsorbents in the literature. 

#### 3.2.4. Effect of Coexisting Ions on Pd(II) Adsorption

**Figure 6:**
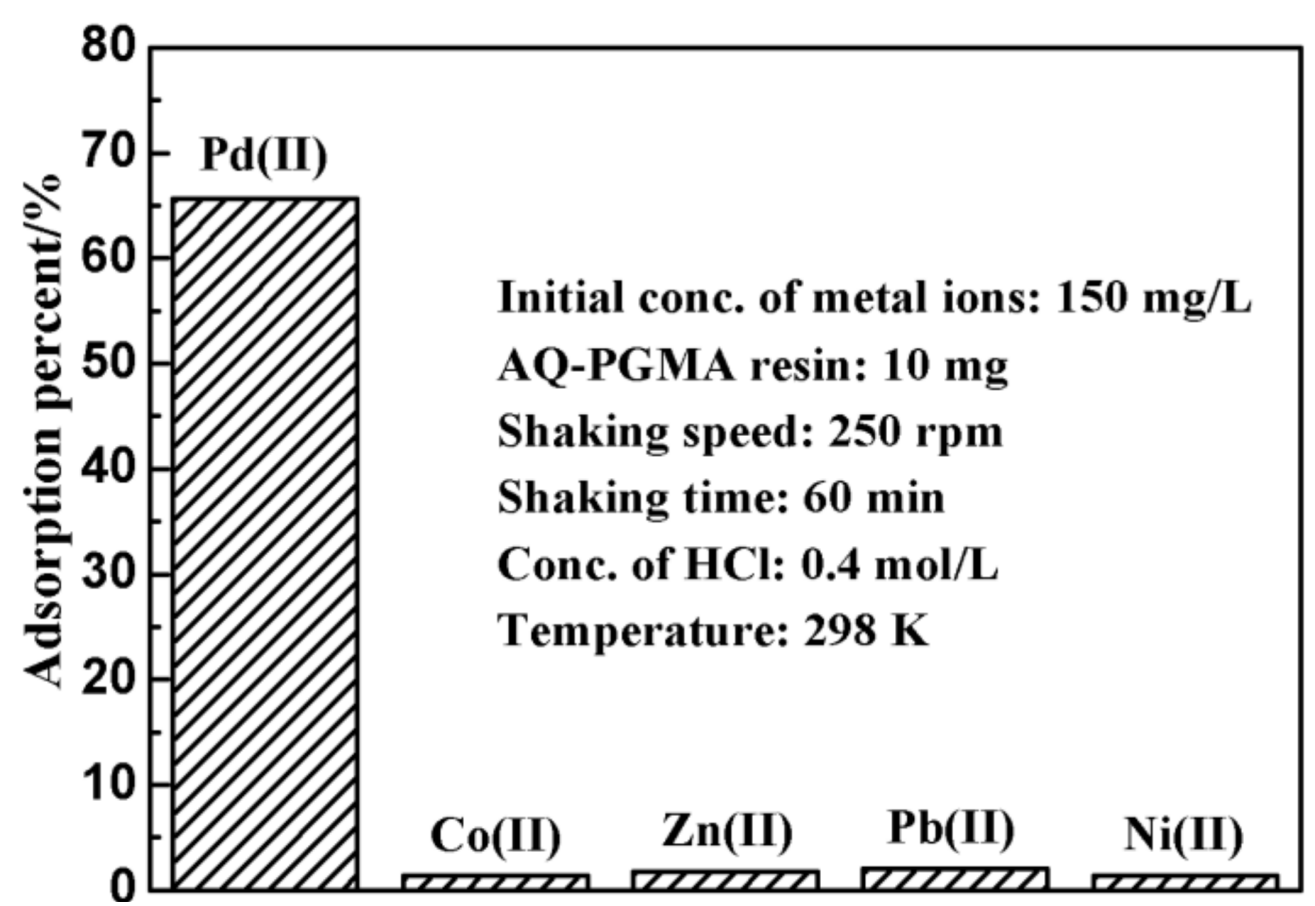
Effect of coexisting ions on the adsorption of Pd(II) by AQ-PGMA.

Adsorption of Pd(II) from coexisting ions solution, including Co.(II), Zn(II), Pb(II) and Ni(II), was studied at fixed adsorption conditions: HCl concentration was 0.4 mol/L; adsorption time was 60 min and the initial concentration of Pd(II), Co.(II), Zn(II), Pb(II) and Ni(II) was 150 mg/L. Figure 6 shows that the adsorption percentages of Co.(II), Zn(II), Pb(II) and Ni(II) were 1.35%, 1.81%, 2.02% and 1.42%, respectively, while the adsorption percentage of Pd(II) was 65.67%. This indicates that AQ-PGMA has selectivity for Pd(II).

#### 3.2.5. Effect of Reused Cycles Of AQ-PGMA on Pd(II) Adsorption

**Figure 7:**
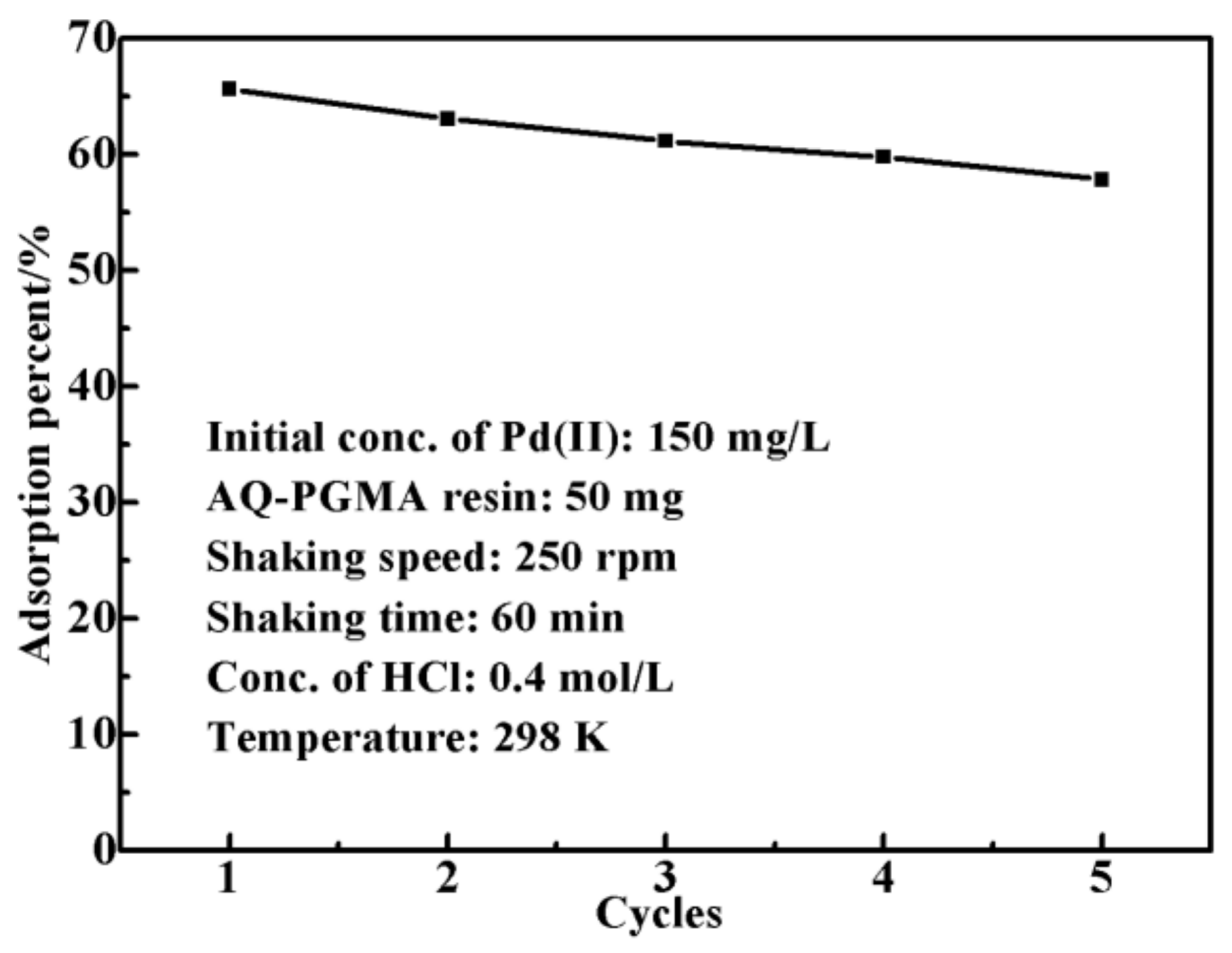
Effect of reused cycles on the recovery of Pd(II) after successive cycles of adsorption/desorption.

The desorption experiments were carried out with a 1 mol/L thiourea solution containing 0.1 mol/L HCl. After desorption, the AQ-PGMA is reused for the adsorption experiment. Figure 7 shows the reused results after 5 successive cycles. Obviously, the effect of the reused cycles on the adsorption percentage of Pd(II) on AQ-PGMA is very small and the maximum desorption rate of 98% can be reached. This indicates that the AQ-PGMA can be reused in the recovery of Pd(II) from aqueous solution for several cycles. Table 6 shows the regeneration efficiency of AQ-PGMA against other sorbents reported in the literature. AQ-PGMA has a higher regeneration efficiency compared to many adsorbents in the literature. 

### 3.3. Adsorption Mechanism of Pd(II) Onto AQ-PGMA

**Figure 8:**
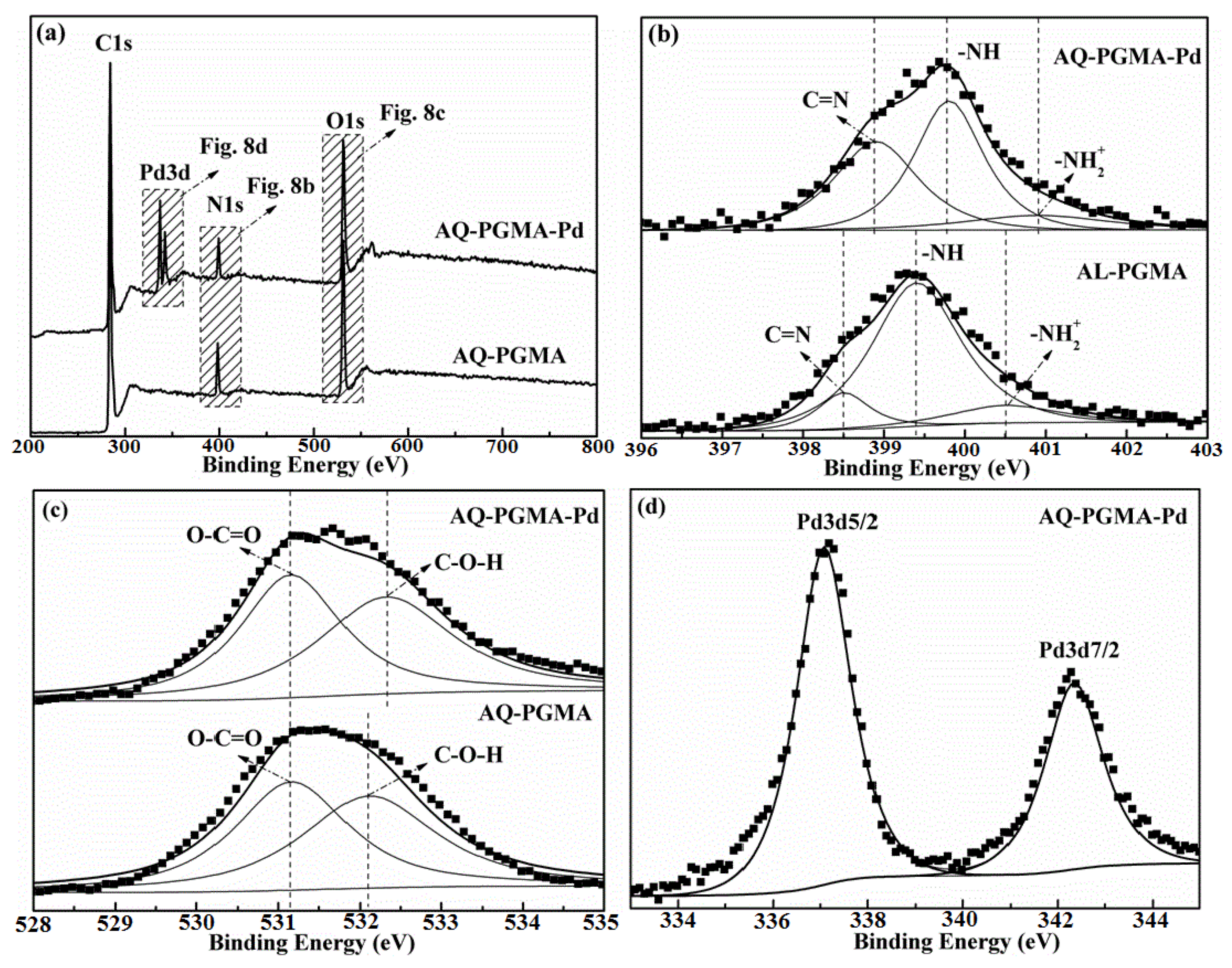
XPS survey scan (**a**), N 1s spectra (**b**), O 1s spectra (**c**) of AQ-PGMA and AQ-PGMA-Pd and Pd 3d spectra (**d**) of AQ-PGMA-Pd.

Figure 8 shows the XPS spectra of AQ-PGMA before and after adsorption of Pd(II) (AQ-PGMA-Pd). The survey scan of AQ-PGMA-Pd presents the Pd 3d peak, due to the adsorption of Pd(II) on AQ-PGMA (Figure 8a). Figure 8b shows the N 1s peaks of AQ-PGMA and AQ-PGMA-Pd. For AQ-PGMA, the N 1s peaks at 398.5, 399.3 and 400.5 eV were attributed to the C=N, –NH and –NH_2_^+^ bonds, respectively. The N 1s peaks moved to 398.9, 399.8 and 401.0 eV after adsorption of Pd(II), which came from the chelation interaction of Pd(II) with the amines/hydroxyl groups [19]. In addition, the peak of –NH_2_^+^ is broadened in AQ-PGMA-Pd compared to the AQ-PGMA, which indicates that the amino groups are protonated and interact with Pd(II) by ion exchange [19]. Figure 8c shows the O 1s peaks of AQ-PGMA and AQ-PGMA-Pd. The peaks of 531.18 and 532.08 eV correspond to the O–C=O and C–O–H bonds in AQ-PGMA. The C–O–H peaks moved to 532.38 eV after adsorbed Pd(II) because the C–O–H was coordinated with Pd [19]. Figure 8d shows the Pd 3d peaks of AQ-PGMA-Pd. The Pd 3d peaks are located at 337.1 and 342.4 eV, which are higher than those of free Pd(II) (335.9 and 341.1 eV) in solution, suggesting that Pd(II) received electrons and transformed into PdCl_4_^2−^ during the adsorption process [32].

**Figure 9:**
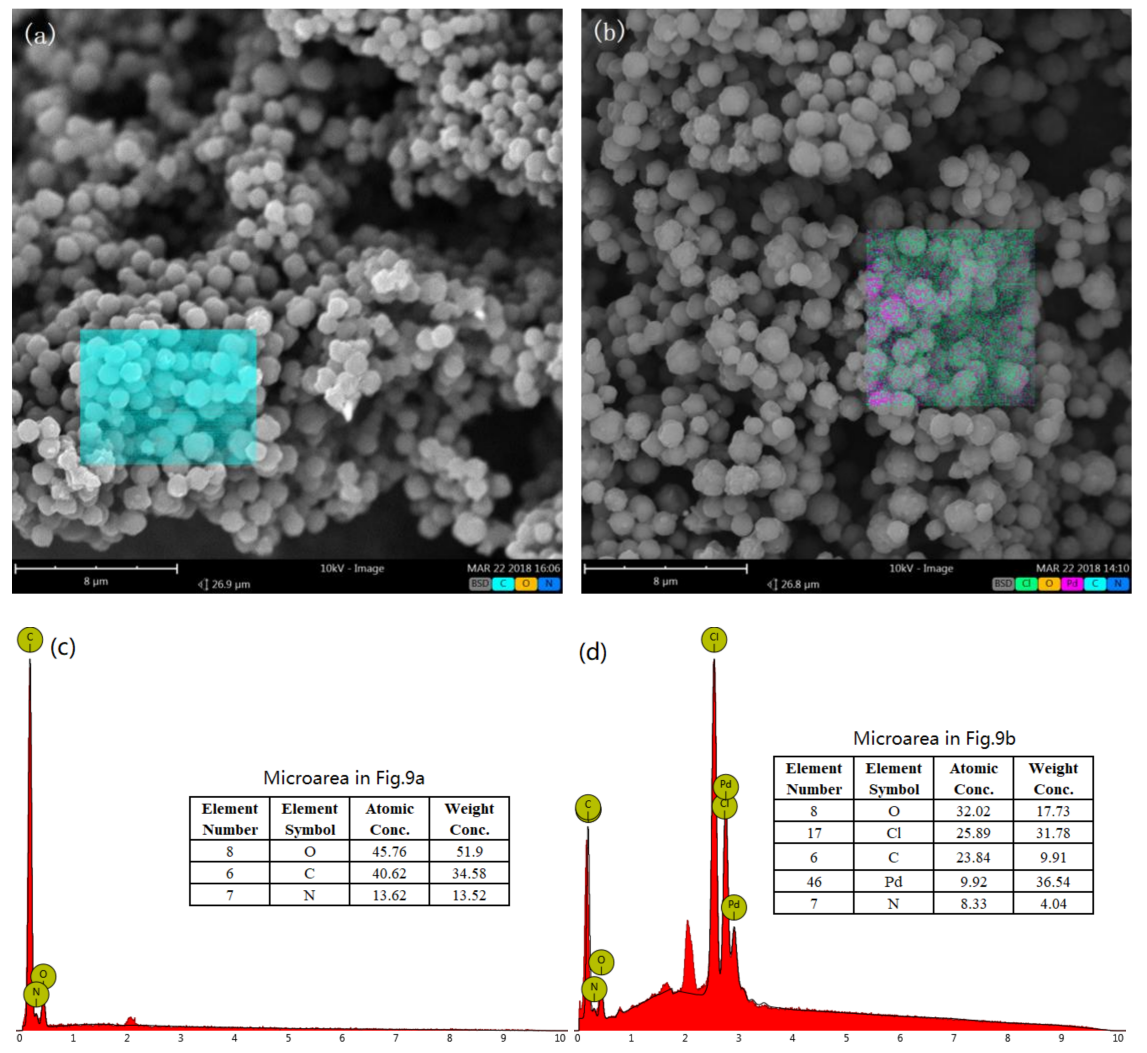
Scanning electron microscopy with energy dispersive spectroscopy (SEM-EDS) analysis of AQ-PGMA and AQ-PGMA-Pd: (**a**) and (**b**) SEM micrographs; (**c**) and (**d**) EDS spectrums.

In order to further investigate the adsorption mechanism of Pd(II) onto AQ-PGMA, scanning electron microscopy with energy dispersive spectroscopy (SEM-EDS) was used to analyze the AQ-PGMA and AQ-PGMA-Pd, the results are shown in Figure 9a–d. It can be seen from Figure 9a,b that the morphology of AQ-PGMA and AQ-PGMA-Pd are microspheres and the diameters are in the range of 0.1 to 1 nm. In addition, the specific surface area and pore volume of AQ-PGMA are 1.969 × 10^4^ and 0.007 cm^3^/g, respectively, which indicates that AQ-PGMA has a great specific surface area. Combined with Figure 4a–d, it can be seen that the high absorption rate of Pd(II) on AQ-PGMA is caused by the great specific surface area of AQ-PGMA so that more Pd(II) can easily access the adsorption site [45]. Figure 9c shows that C, O and N appear on the surface of the AQ-PGMA. The new elements of Cl and Pd appear in AQ-PGMA-Pd after adsorption of the Pd(II) (Figure 9d).

**Scheme 2:**
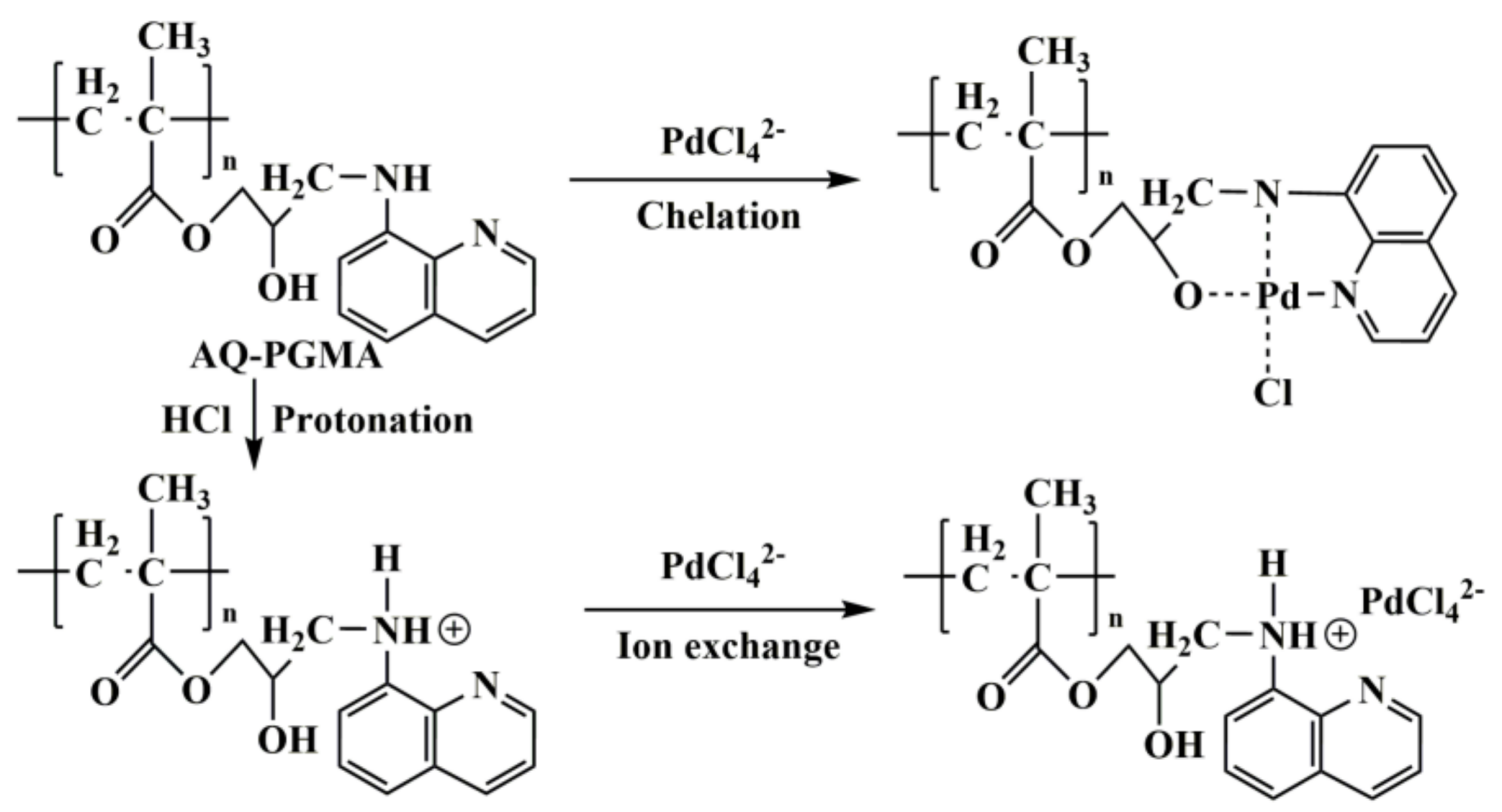
Mechanism of the adsorption of Pd(II) onto AQ-PGMA.

Based on the analysis of the effect of the HCl concentration and adsorption kinetics, it can be found that the adsorption of Pd(II) is related to the ion exchange and chemical action. The adsorption mechanism of Pd(II) onto AQ-PGMA is summarized in Scheme 2. For the chemical action, the amines and hydroxyl groups on the AQ-PGMA chelated directly with PdCl_4_^2–^ by providing electrons. For the ion exchange, the protonated amino group adsorbed PdCl_4_^2–^. 

## 4. Conclusions

The 8-aminoquinoline-grafted poly(glycidyl methacrylate) has been successfully synthesized for the efficient recovery of Pd(II) from highly acidic aqueous solutions. The recovery of Pd(II) by AQ-PGMA was researched by batch test and the sorption results disclosed that the recovery of Pd(II) was closely related to the concentration of hydrochloric acid. The highest adsorbing capacity of Pd(II) is 267.90 mg/g on AQ-PGMA when the concentration of Cl^–^ is 0.4 mol/L or over. The findings indicated that Pd(II) adsorption occurred mainly through the pseudo-second-order model. Sorption isotherm followed the Hill model and the number of Pd(II) per site was 1.45. AQ-PGMA can be simultaneously revived into its initial form for the next procedure and has no significant loss of its adsorption performance. AQ-PGMA presented the highest selectivity for Pd(II) from the coexisting ions solution including Co(II), Zn(II), Pb(II) and Ni(II). The chelation and ion exchange between PdCl_4_^2–^ and amines/hydroxyl groups are the main driving force of adsorption. So, AQ-PGMA has great potential for the efficient recovery of Pd(II) from highly acidic aqueous solutions.

## Figures and Tables

**Table 1:** Equations of adsorption kinetic models.

Kinetic models	Equations
Pseudo-first-order	$\ln\left(q_{e,1}-q_{t,1}\right)=-k_{1}t+lnq_{e,1}$
Pseudo-second-order	$\frac{t}{q_{t,2}}=\frac{t}{q_{e,2}}+\frac{1}{k_{2}q_{e,2}^{2}}$
Intra-particle diffusion	$q_{t}=k_{i}t^{1/2}+C$

**Table 2:** Parameters of kinetic models on the adsorption of Pd(II) by AQ-PGMA.

Pseudo-first-order	*k*_1_ (g/(mg·min))		*q*_e,1_ (mg/g)	*R*^2^
	0.01		14.88	0.5125
Pseudo-second-order	*k*_2_ (g/(mg·min))		*q*_e,2_ (mg/g)	*R*^2^
	1.54 × 10^−3^		151.29	0.9993
Intra-particle diffusion	Stage 1	*k*_i1_ (mg/(g·min^1/2^))	*C*_1_	*R*_1_^2^
		12.71	52.69	0.9495
	Stage 2	*k*_i2_ (mg/(g·min^1/2^))	*C*_2_	*R*_2_^2^
		−0.19	151.26	0.7828
Experimental data	*q*_e_ (mg/g)			
	148.60			

**Table 3:** The equilibrium time and kinetic models rate constant of AQ-PGMA and other adsorbents in literature.

Adsorbents	Equilibrium time/h	Kinetic rate constant (*k*_2_)/g/(mg·min)	References
TCS	5	5.05 × 10^−4^	[30]
PAN-TU-GA	6	2.10 × 10^−6^	[31]
GCC	5	1.364 × 10^−5^	[32]
Diaion WA21J	10	1.17 × 10^−5^	[34]
AQ-PGMA	1	1.54 × 10^−3^	This work

**Table 4:** Parameters of isotherm models on the adsorption of Pd(II) by AQ-PGMA.

Freundlich	*K*_F_		*n*_F_		*R*^2^
	79.24		5.06		0.829
Langmuir	*K*_L_		*q*_m_ (mg/g)		*R*^2^
	0.02		292.14		0.9807
Hill	*C*_1/2_ (mg/L)	*N*_M_ (mg/g)	*n*	*q*_0_ (mg/g)	*R*^2^
	45.88	188.469	1.45	273.28	0.9988
Experimental data	*q*_0_ (mg/g)				
	267.90				

**Table 5:** Sorption capacity of AQ-PGMA against other sorbents reported in the literature.

Adsorbents	*q*_m_ (mg/g)	References
polyethylene fibers	221.8	[43]
macroporous resins XAD-7 HP	52.77	[44]
Racomitrium lanuginosum biomass	37.2	[45]
Biopolymer modified activated carbon	43.5	[46]
Chitosan	5.88	[47]
Cross-linked chitosan modified with l-lysine	109.47	[48]
AQ-PGMA	267.90	This work

**Table 6:** Regeneration efficiency of AQ-PGMA against other sorbents reported in the literature.

Adsorbents	Desorption efficiency/%	References
TCS	95	[30]
PAN-TU-GA	93	[31]
GCC	42.35	[32]
AQ-PGMA	98	This work
